# Supplementary material for: HPV infection alters vaginal microbiome through down-regulating host mucosal innate peptides used by Lactobacilli as amino acid sources
Source: Nat Commun. 2022 Feb 28;13:1076. doi: 10.1038/s41467-022-28724-8 (PMC8885657; doi:10.1038/s41467-022-28724-8)
Supplement: Supplementary file 1 — Supplementary Information [file 41467_2022_28724_MOESM1_ESM.pdf]

Supplementary information

HPV infection alters vaginal microbiome through down-regulating host mucosal innate peptides used by *Lactobacilli* as amino acid sources

Alizee Lebeau[1*], Diane Bruyere[1*], Patrick Roncarati[1], Paul Peixoto[2,3], Eric Hervouet[2,3], Gael Cobraiville[4], Bernard Taminiau[5], Murielle Masson[6], Carmen Gallego[7], Gabriel Mazzucchelli[8], Nicolas Smargiasso[8], Maximilien Fleron[8,9], Dominique Baiwir[8,9], Elodie Hendrick[1], Charlotte Pilard[1], Thomas Lerho[1], Celia Reynders[1], Marie Ancion[1], Roland Greimers[10], Jean-Claude Twizere[11], Georges Daube[5], Geraldine Schlecht-Louf[7], Françoise Bachelerie[7], Jean-Damien Combes[12], Pierrette Melin[13], Marianne Fillet[4], Philippe Delvenne[1,10], Pascale Hubert[1] and Michael Herfs[1]

[1]Laboratory of Experimental Pathology, GIGA-Cancer, University of Liege, 4000 Liege, Belgium
[2]INSERM, EFS BFC, UMR 1098, Interactions Hôte-Greffon-Tumeur/Ingénierie Cellulaire et Génique, University of Bourgogne Franche-Comté, 25000 Besançon, France
[3]EPIGENEXP platform, University of Bourgogne Franche-Comté, 25000 Besançon, France
[4]Laboratory for the Analysis of Medicines, Center for Interdisciplinary Research on Medicines (CIRM), University of Liege, 4000 Liege, Belgium
[5]Department of Food Sciences-Microbiology, Fundamental and Applied Research for Animals and Health (FARAH), Faculty of Veterinary Medicine, University of Liege, 4000 Liege, Belgium
[6]Ecole Supérieure de Biotechnologie Strasbourg, UMR 7242, CNRS, University of Strasbourg, 67412 Illkirch, France
[7]INSERM UMR 996, Inflammation Microbiome and Immunosurveillance, University of Paris-Saclay, 92140 Clamart, France
[8]Laboratory of Mass Spectrometry, Department of Chemistry, University of Liege, 4000 Liege, Belgium
[9]GIGA Proteomic Facility, University of Liege, 4000 Liege, Belgium
[10]Department of Pathology, University Hospital Center of Liege, 4000 Liege, Belgium
[11]Laboratory of Signaling and Protein Interactions, GIGA-Molecular Biology of Diseases, University of Liege, 4000 Liege, Belgium
[12]Infections and Cancer Epidemiology Group, International Agency for Research on Cancer, World Health Organization, 69372 Lyon, France
[13]Department of Clinical Microbiology, University Hospital Center of Liege, 4000 Liege, Belgium

*These authors contributed equally: Alizee Lebeau and Diane Bruyere.

Authors to whom correspondence, proofs and reprint requests should be sent:

Michael Herfs, PhD
Laboratory of Experimental Pathology, GIGA-Cancer, B23 +4
University of Liege
4000 Liege
Belgium
Email: M.Herfs@uliege.be

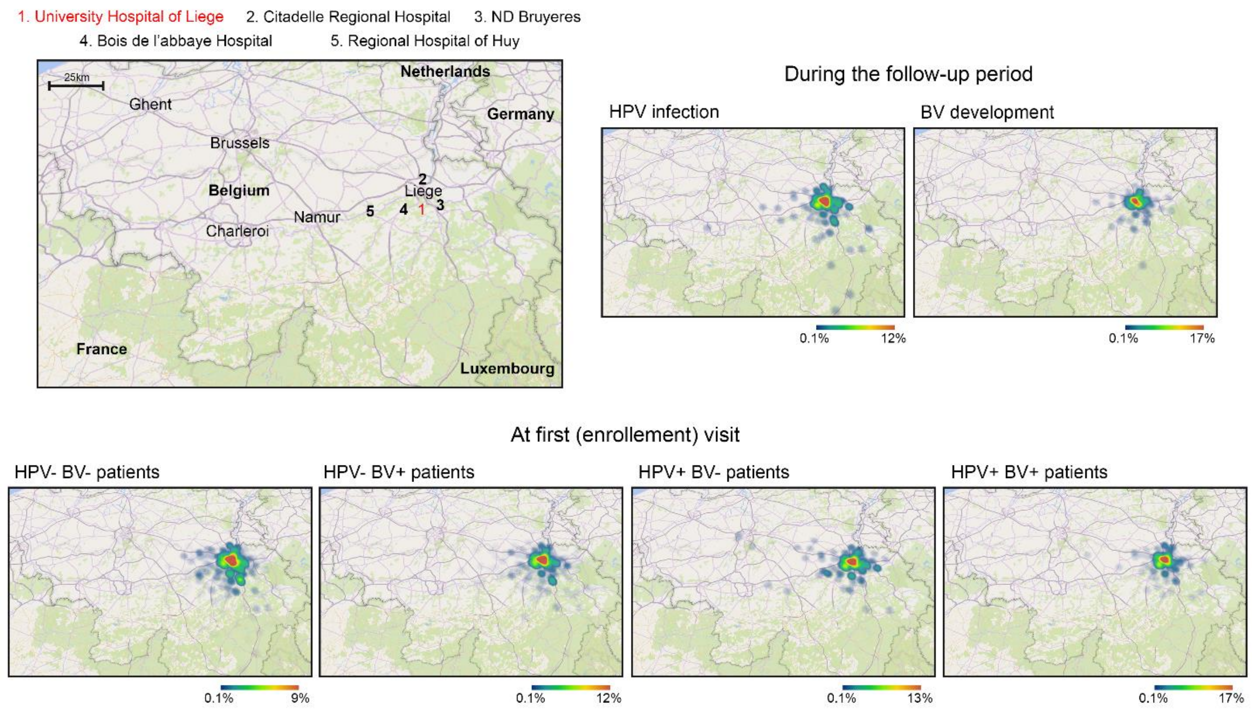

Supplementary Figure 1. Geographic distribution of patients (6,045/6,085, 99.3%) evaluated in the present retrospective cohort analysis. Of note, the large majority of patients lived within a 50km radius around Liege (Belgium) and no obvious difference in terms of geographic repartition of patients was noticed between the defined groups. Source data are provided as a Source Data file.

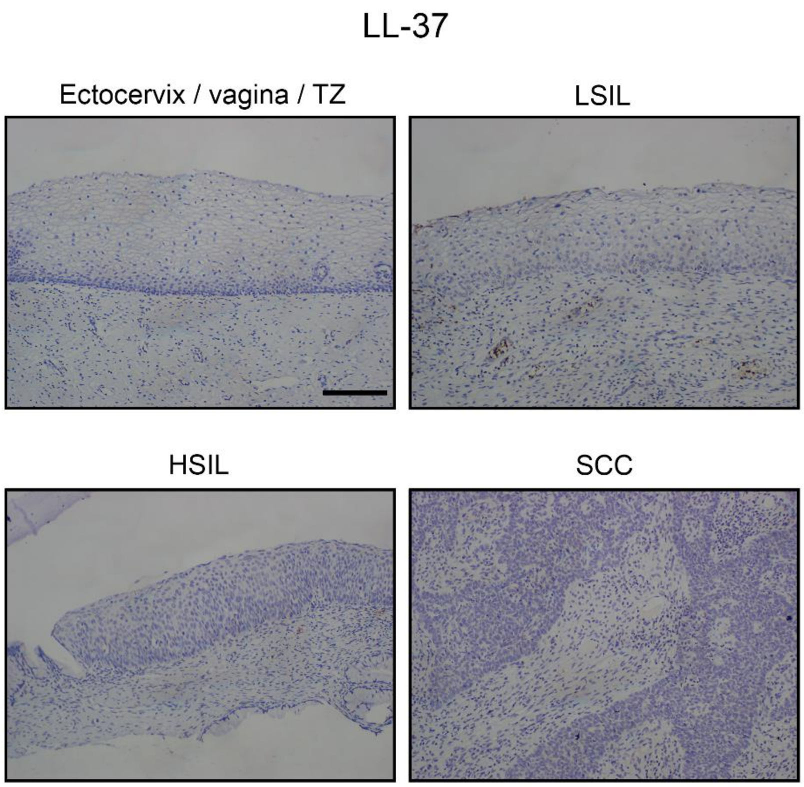

Supplementary Figure 2. Representative examples of anti-LL-37 immunoreactivity displayed by both normal squamous epithelium (ectocervix/vagina) and HPV-positive (pre)neoplastic lesions. Note the absence of LL-37 expression in epithelial compartment. Twenty HPV-negative tissue specimens and 45 HPV-related (pre)neoplastic lesions (LSIL, n=15; HSIL, n=15; SCC, n=15) were stained. TZ: transformation zone; SIL: squamous intraepithelial lesion; SCC: squamous cell carcinoma. The scale bar represents 100 µm.

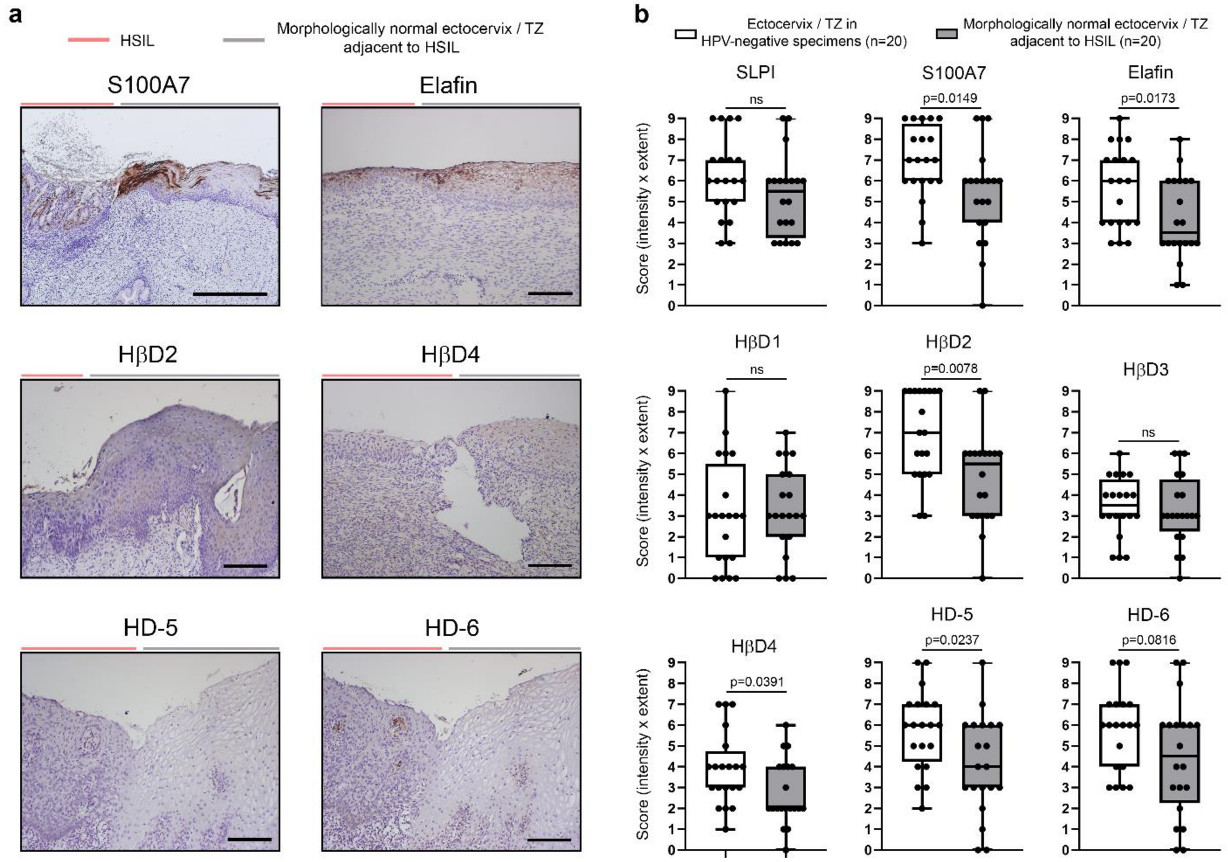

Supplementary Figure 3. Morphologically normal squamous epithelia adjacent to HPV-positive (pre)neoplastic lesions display a reduced expression of several innate (antimicrobial) peptides. a Representative pictures of morphologically normal ectocervix / TZ adjacent to CIN 2/3 stained for S100A7, elafin, HβD2, HD-5 and HD-6. **b** Semi-quantitative evaluation of innate peptide expression (intensity and extent of the immunostaining) in normal squamous epithelia from HPV-negative specimens (n=20, individual data from Figure 2F) and morphologically normal tissues adjacent to CIN 2/3 (n=20). The significantly reduced anti-S100A7, anti-elafin, anti-HβD2, anti-HβD4 and anti-HD-5 immunoreactivities should be noticed. Box limits: 25th to 75th percentiles; line: median; whiskers: minimum to maximum. The scale bar represents 100 µm. *P* values were determined using two-sided Mann-Whitney test. ns: not significant (p<0.1). Source data are provided as a Source Data file.

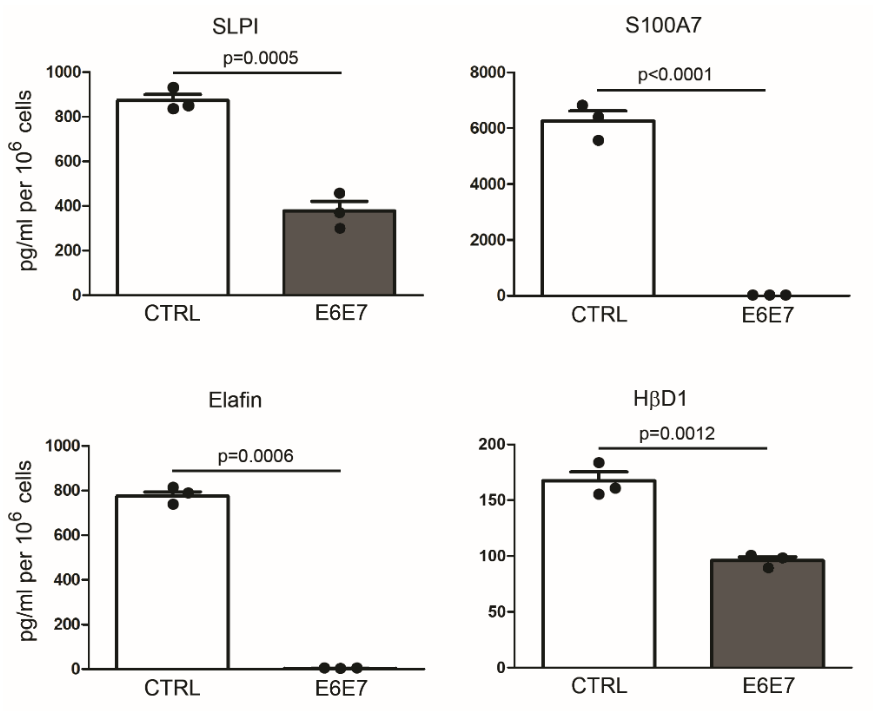

Supplementary Figure 4. Secretion of defensin-like peptides (SLPI, elafin, S100A7) and HβD1 analyzed by ELISA. Note the drastic reduced secretion of these four so called "constitutive" peptides in case of HPV16 E6E7 transduction. Results represent the means ± SEM of three independent experiments. *P* values were determined using two-sided unpaired *t*-tests. Source data are provided as a Source Data file.

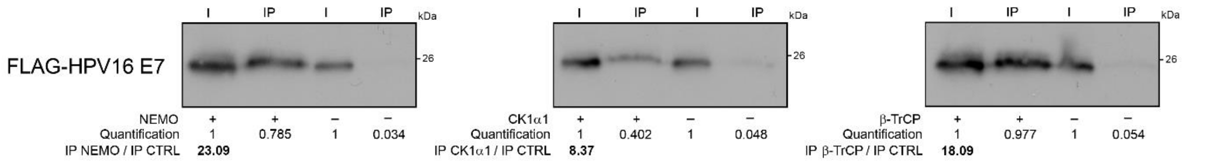

Supplementary Figure 5. Validation of HPV16 E7-NEMO, E7-CK1 and E7-β-TrCP interactions by co-IP in the inverse direction. In contrast to the results presented in the main Figures 3 and 4, anti-NEMO, anti-CK1α1 and anti- β-TrCP antibodies were used for the immunoprecipitation step. FLAG-E7 was then detected. Note the clear enrichments supporting the bindings highlighted by GPCA. Each co-IP analysis was independently performed twice and one representative experiment is shown. Source data are provided as a Source Data file.

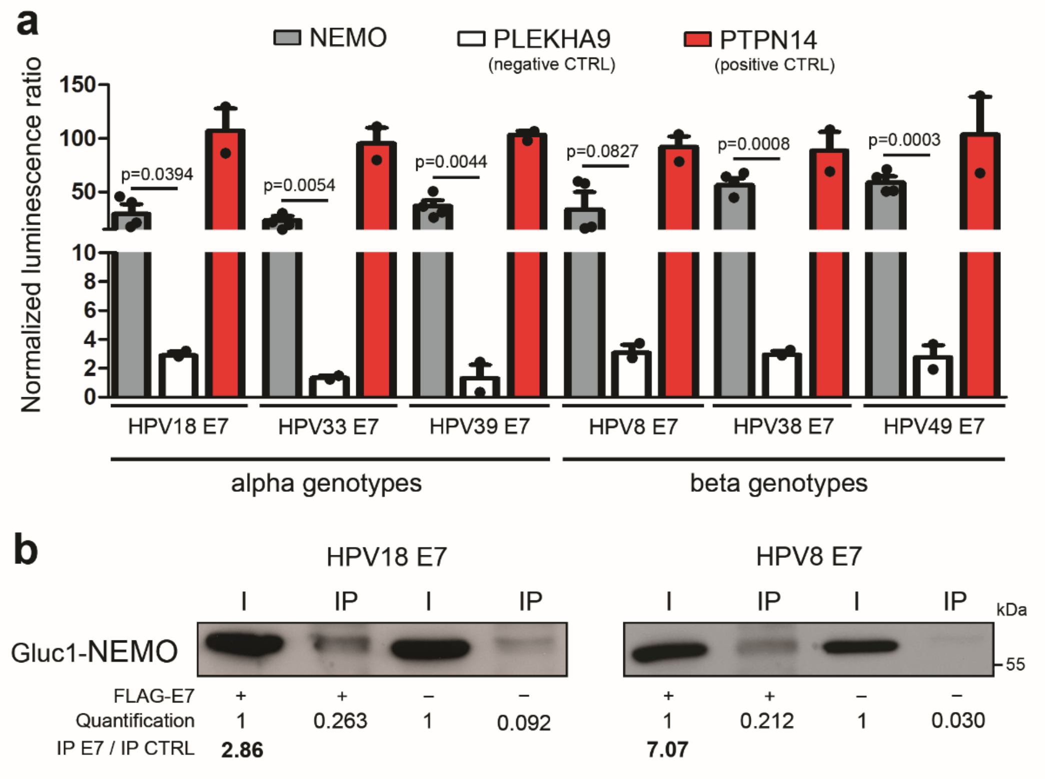

Supplementary Figure 6. Binding of NEMO with E7 oncoprotein from six different HPV genotypes (high-risk alpha: HPV16, 18, 33 and 39; beta: HPV8, 38 and 49) analyzed by GPCA (a) and confirmed by co-IP (b). For the GPCA experiments, PTPN14 and PLEKHA9 were used as positive and negative control, respectively. Results represent the means ± SEM of two (PTPN14 and PLEKHA9) or four (NEMO) independent experiments. Each co-IP analysis was independently performed twice and one representative experiment is shown. *P* values were determined using two-sided unpaired *t*-tests. Source data are provided as a Source Data file.

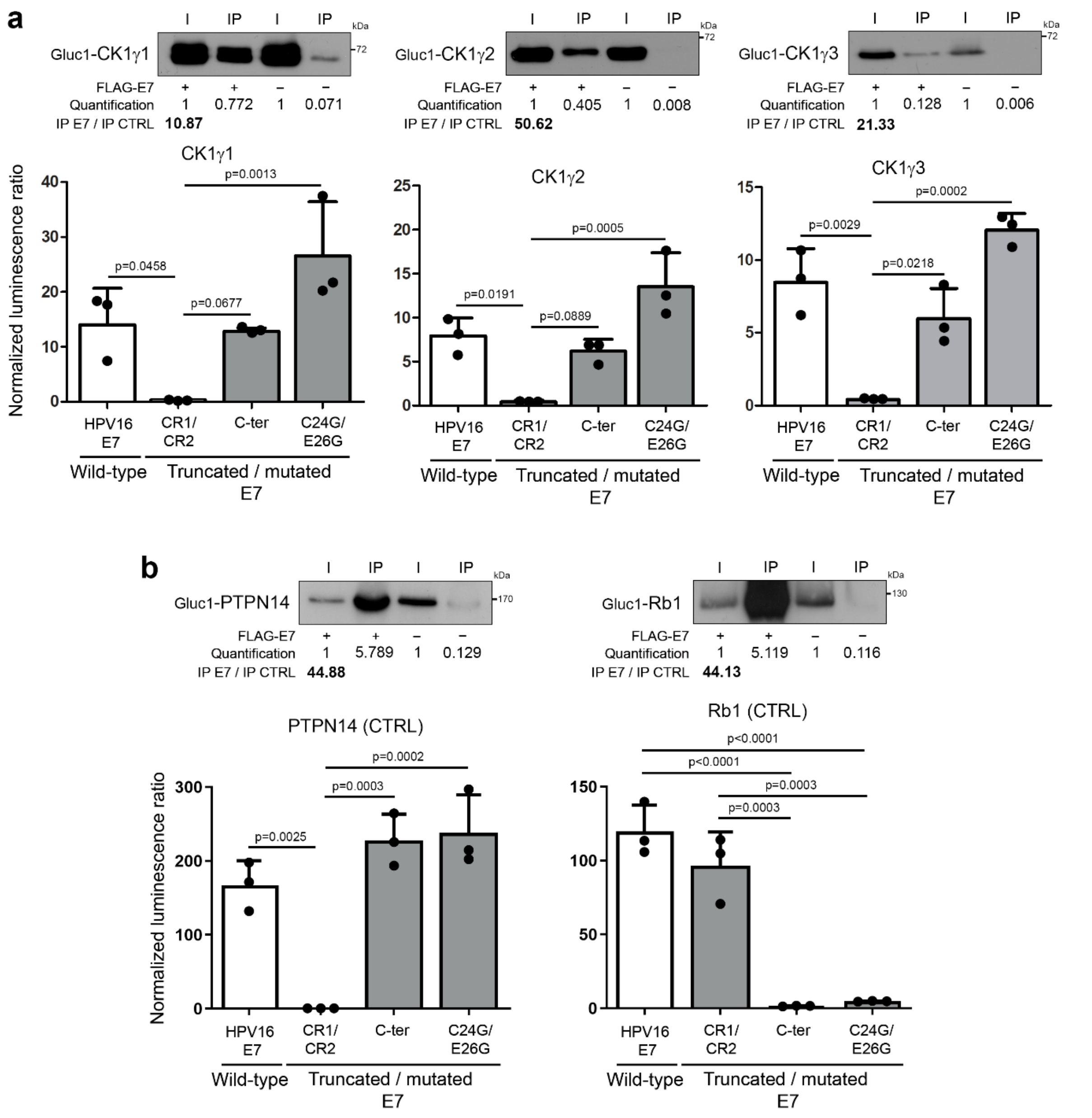

Supplementary Figure 7. Interaction between gamma isoforms (γ1-3) of CK1 and HPV16 E7 oncoprotein. a The bindings were validated by both Co-IP and GPCA using the truncated/mutated forms of E7. Of note, the GPCA signal was drastically reduced with the CR1+CR2 construct, supporting that CK1γ1, CK1γ2 and CK1γ3 interact with the C-terminal region of E7. Results represent the means ± SEM of three independent experiments. **b** PTPN14 and Rb1 were systematically used as positive control for binding to the C-terminal region and the LxCxE motif (included in the N-terminal half) of E7, respectively. Results represent the means ± SEM of three independent experiments. Each co-IP analysis was independently performed twice and one representative experiment is shown. *P* values were determined using one-way ANOVA followed by Bonferroni post-hoc test. Source data are provided as a Source Data file.

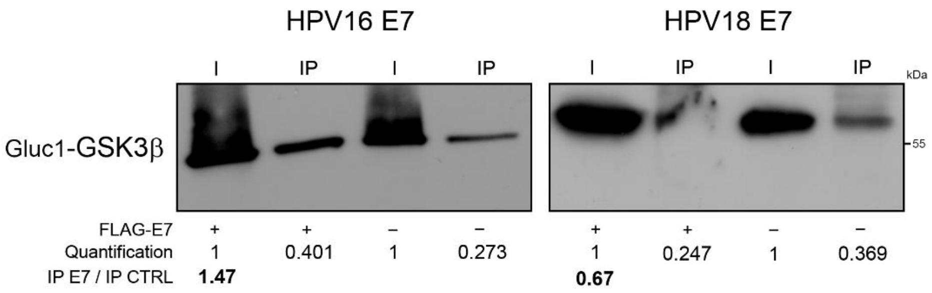

Supplementary Figure 8. Co-IP of GSK3β with E7 oncoprotein from HPV16 and 18. Note the absence of clear enrichment suggesting that GSK3β represents a false positive of the GPCA screening method. Each co-IP analysis was independently performed twice and one representative experiment is shown. Source data are provided as a Source Data file.

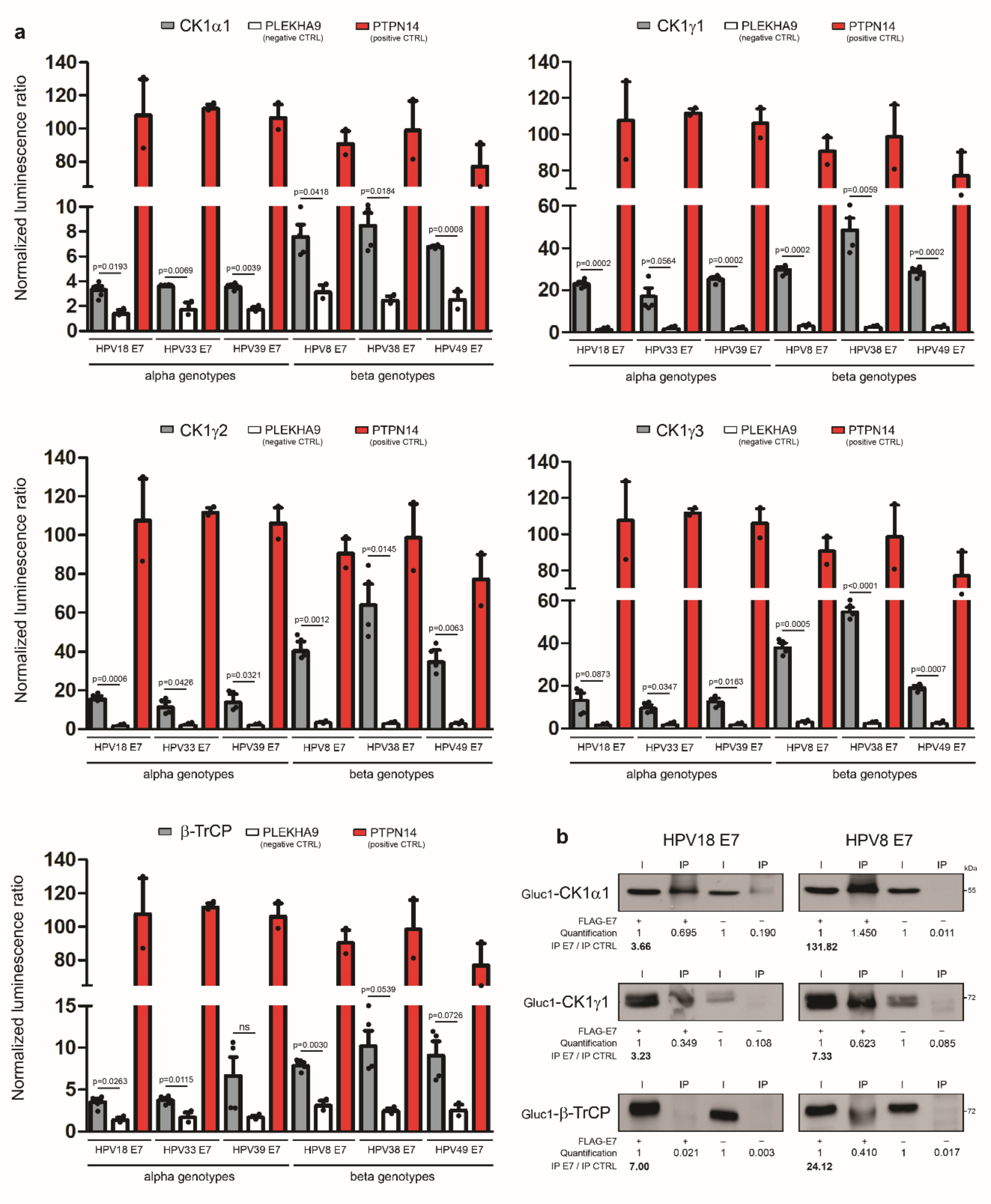

Supplementary Figure 9. Binding of alpha and gamma isoforms of CK1 and β-TrCP with E7 oncoprotein from six different HPV genotypes (high-risk alpha: HPV16, 18, 33 and 39; beta: HPV8, 38 and 49) analyzed by GPCA (a) and confirmed by co-IP (b). For the GPCA experiments, PTPN14 and PLEKHA9 were used as positive and negative control, respectively. Results represent the means ± SEM of two (PTPN14 and PLEKHA9) or four (CK1 isoforms and β-TrCP) independent experiments. Each co-IP analysis was independently performed twice and one representative experiment is shown. *P* values were determined using two-sided unpaired *t*-tests. ns: not significant (p<0.1). Source data are provided as a Source Data file.

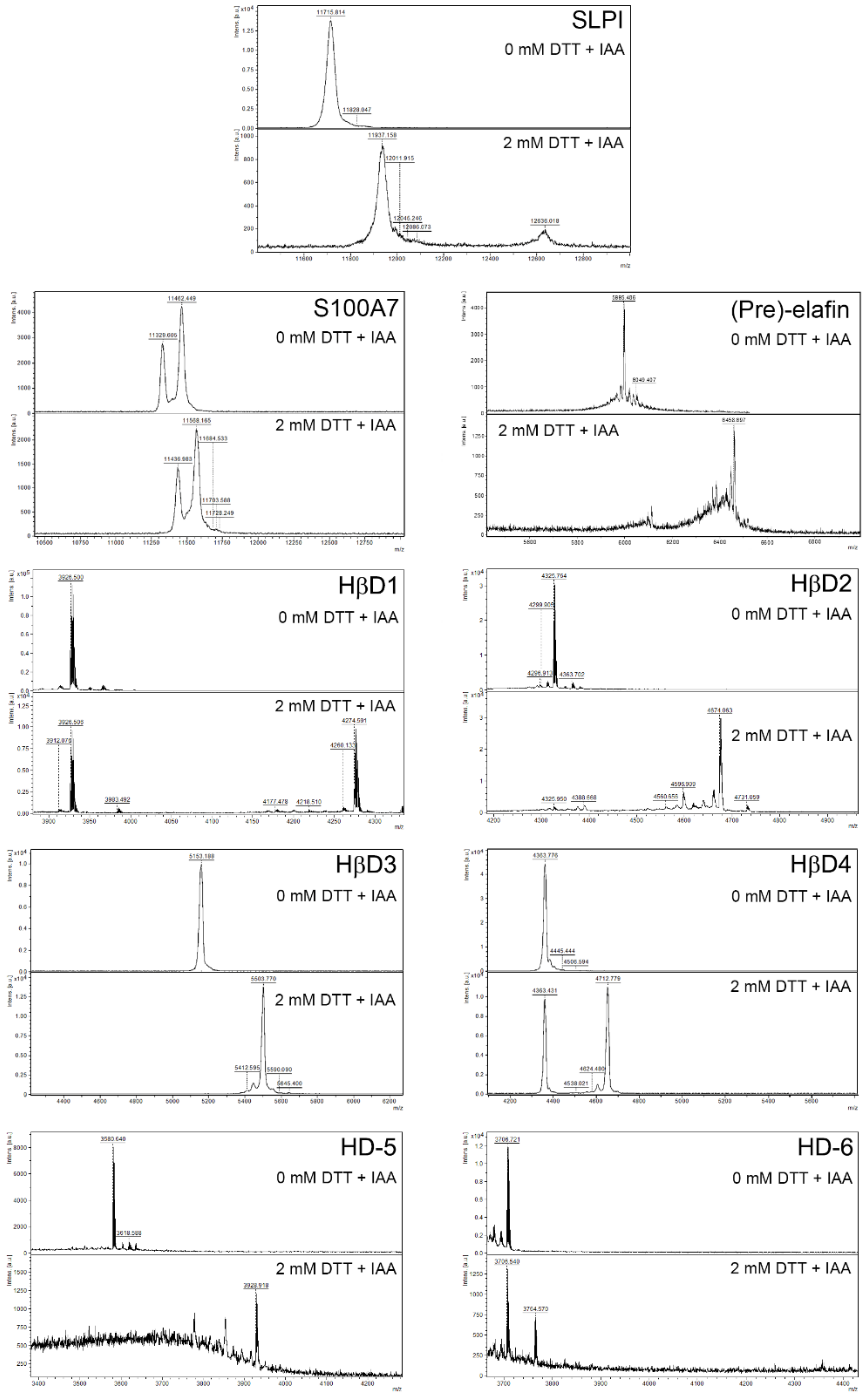

Supplementary Figure 10. MALDI-TOF mass spectra of oxidized innate peptides incubated with or without DTT (2mM) and alkylated with iodoacetamide (IAA). In addition to the chemical reduction of the disulfide bridges, both the synthesis and the purity of each tested peptide was examined.

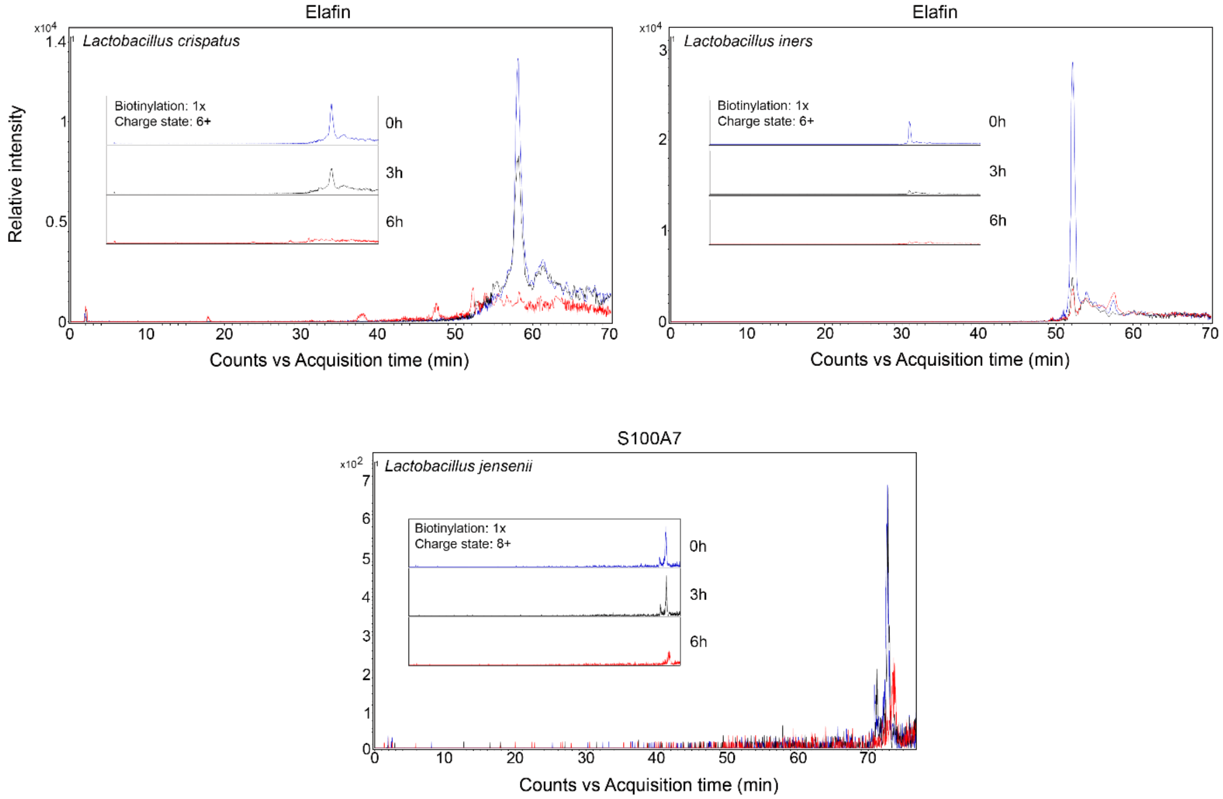

Supplementary Figure 11. Dominant *Lactobacillus* species constituting the vaginal microbiome hydrolyze some innate peptides highly secreted by host mucosa. Note the nearly complete disappearance of native elafin/S100A7 after 6h of incubation with lactic acid bacteria (*L. crispatus*, *L. jensenii* and *L. iners*).

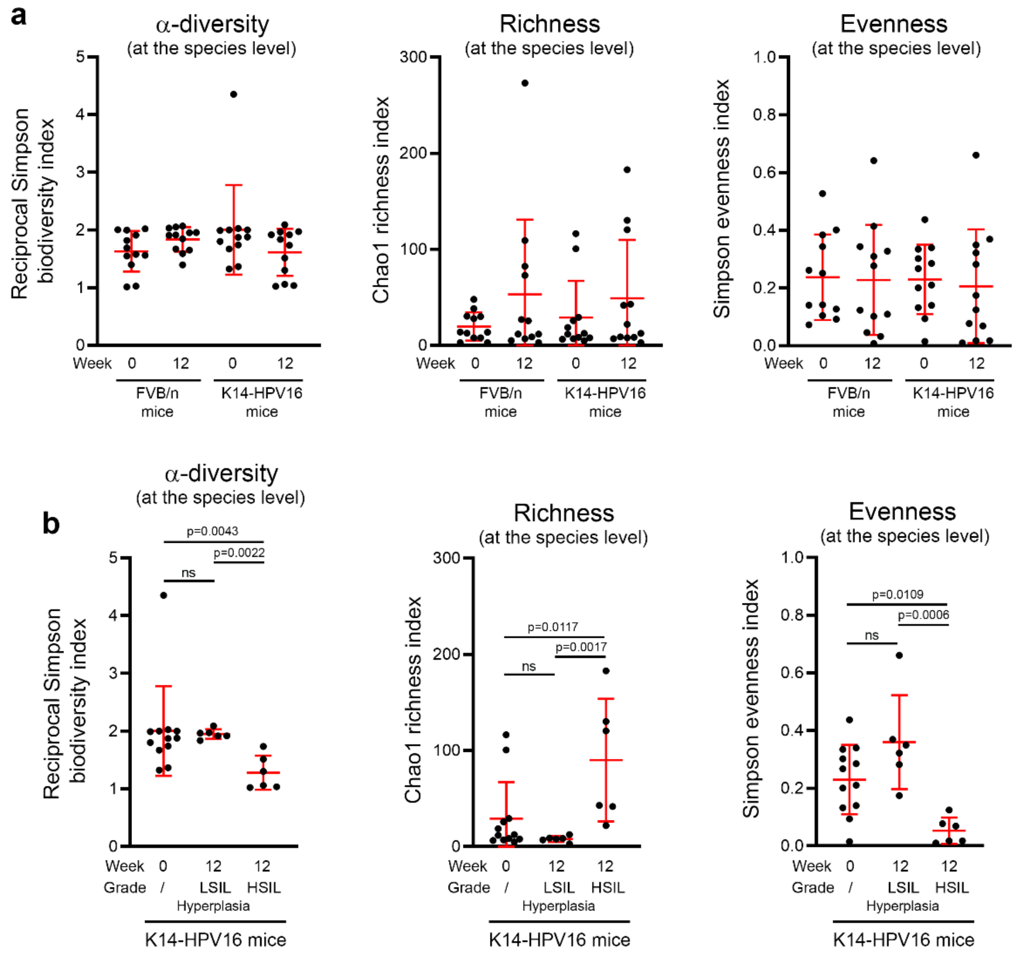

Supplementary Figure 12. Bacterial intrinsic diversity, richness and evenness at the species level. Each individual value in the four defined groups [FVB/n (week 0 *versus* week 12) and K14-HPV16 (week 0 *versus* week 12)] (**a**) or only in K14-HPV16 mice (**b**) are shown (n=12 per group). Significant decreases in bacterial diversity and evenness as well as an increased bacterial richness in K14-HPV16 mice displaying a high-grade squamous intraepithelial lesion (HSIL, n=6) were observed. The means ± SD are represented. *P* values were determined using Kruskal-Wallis test corrected with a two-stage linear step-up procedure of Benjamini, Krieger and Yekutieli. ns: not significant (p<0.1). Source data are provided as a Source Data file.

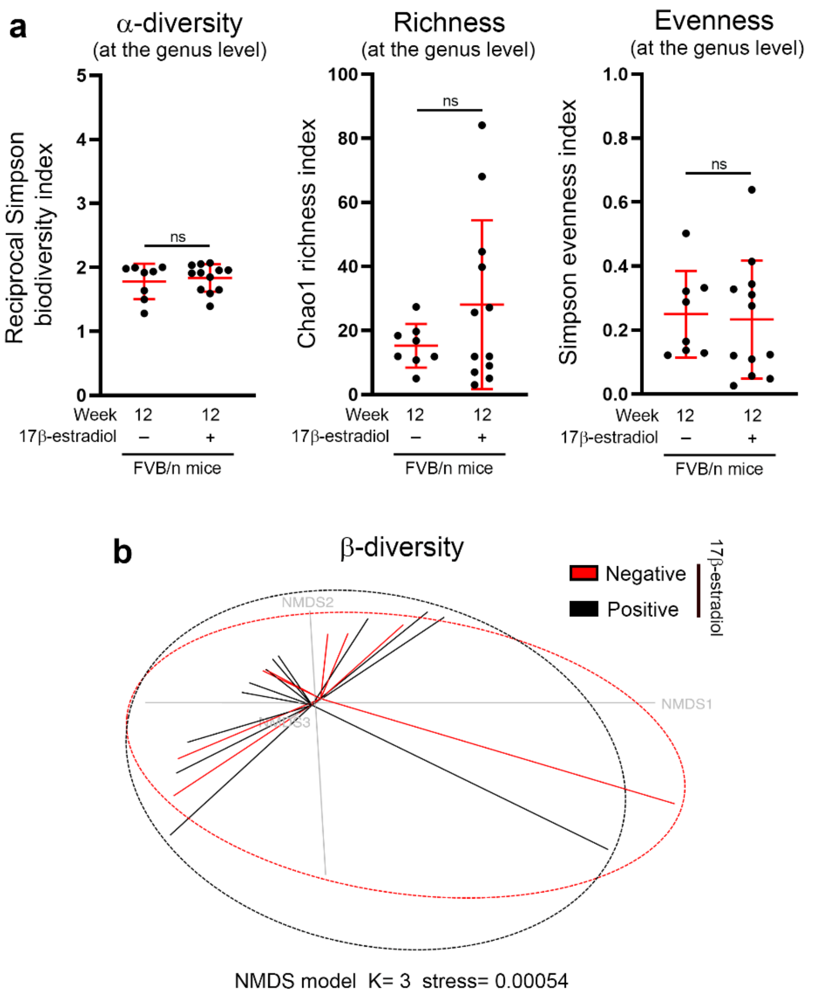

Supplementary Figure 13. Long-term estrogen treatment does not modify the composition of murine vaginal microbiota. a Bacterial α-diversity, richness and evenness (at the genus level) for FVB/n mice treated (n=12) or not (n=8) with 17β-estradiol (1-2 µg/24h) during 12 weeks were compared. The means ± SD are represented. **b** β-diversity of the vaginal microbial profile was visualized using a non-metric multidimensional scaling (NMDS) model. An absence of group clustering was clearly observed. *P* values were determined using Mann-Whitney test. ns: not significant (p<0.1). Source data are provided as a Source Data file.

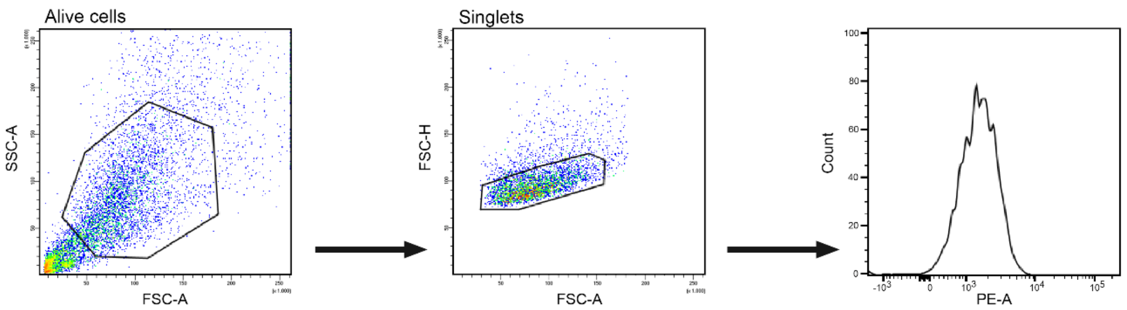

Supplementary Figure 14. Gating strategy used in flow cytometry analysis experiments.

Supplementary Table 1. PCR primers used in the present study

Human qRT-PCR

SLPI	Forward	GCA TCA AAT GCC TGG ATC CT
	Reverse	GCA TCA AAC ATT GGC CAT AAG TC
S100A7	Forward	TTC CCC AAC TTC CTT AGT GCC
	Reverse	GGC GAG GTA ATT TGT GCC CT
Elafin	Reverse	GGA TGA CAT ATG GCT CCA CTC TT
	Forward	TGG CTC CTG CCC CAT TAT C
LL-37	Forward	GAA GGA CGG GCT GGT GAA G
	Reverse	ACC CAG CAG GGC AAA TCT C
HβD1	Forward	GTC GCC ATG AGA ACT TCC TAC C
	Reverse	CAT TGC CCT CCA CTG CTG AC
HβD2	Reverse	GGT CCT TTT CAC CAG CAA GCT
	Forward	TGA TGC CTC TTC CAG GTG TTT
HβD3	Forward	AGT GAC CAA GCA CAC CTT TTC A
	Reverse	CCA AAA ACA GGA AGA GCA AAG C
HβD4	Forward	CCT GTT ACC TGC CTT AAG AGT G
	Reverse	GAA TCC GCA TCA GCC ACA G
HD-5	Forward	GAA TTC AGC ATG GCC ACC TGC TAT TGC
	Reverse	GCG GCC GCT CAG CGA CAG CAG AGT CTG TA
HD-6	Forward	GCT TTG GGC TCA ACA AGG GCT TTC
	Reverse	GAC ACA CGA CAG TTT CCT TCT AGG TCA TA
HPRT	Forward	TGA CAC TGG CAA AAC AAT GCA
	Reverse	GGT CCT TTT CAC CAG CAA GCT
GAPDH	Forward	ACC AGG TGG TCT CCT CTG AC
	Reverse	TGC TGT AGC CAA ATT CGT TG
18S	Forward	CTT CCA CAG GAG GCC TAC AC
	Reverse	CGC AAA ATA TGC TGG AAC TTT
TBP	Forward	TTC TGG GAA AAT GGT GTG C
	Reverse	CCC ACC ATA TTC TGA ATC TT
E7 HPV16	Forward	AGC TCA GAG GAG GAG GAT GAA
	Reverse	GGT TAC AAT ATT GTA ATG GGC TC

Mouse qRT-PCR

SLPI	Forward	GGC CTT TTA CCT TTC ACG GTG
	Reverse	GGC TCC GAT TTT GAT AGC ATC AT
S100A7	Forward	TTG GAT AGT GTG CCT CGC TT
	Reverse	CCG GAA CAG CTC TGT GAT GTA
MβD1	Forward	AGG TGT TGG CAT TCT CAC AAG

	Reverse	GCT TAT CTG GTT TAC AGG TTC CC
MβD4	Reverse	CTG TCT CCA CTT GCA GCC TT
	Forward	ATC TGT CGA AAA GCG GTA GGG
MβD12	Forward	GCA GAG AGG TGT TCT ATT TTG GT
	Reverse	CAT GTC TCG CAC ACA GCA AT
MβD14	Forward	TCC AGG GGA CGC ATT CCT A
	Reverse	CAA GGC AGT TAA GTA CAG CAC A
MβD15	Forward	TGG AAA GAG CAA GAG CAA CCA
	Reverse	CAG GTG CCA CAA CAA AAG GA
HPRT	Forward	AGT CCC AGC GTC GTG ATT AG
	Reverse	TTT CCA AAT CCT CGG CAT AAT GA
GAPDH	Forward	CTC TCT TGC TCA GTG TCC TTG CT
	Reverse	GGT GGA CCT CAT GGC CTA CA

Bisulfite genomic sequencing

CDH1 (promoter)	Forward	TGG GTG AAA GAG TGA GAT TTT AT
	Reverse	CAA ACT CAC AAA TAC TTT ACA ATT C

ChIP

Elafin (promoter)		
−2 kb	Forward	TCT TTA GGA GTG CAA TTG CTG A
	Reversed	ATT TTA GAT TTG AAT GGG TTG TTT
NF-κB −153	Forward	TGG GAC AAT CAG AGA TGA TGT GA
	Reversed	GGG CGG GTC TGT GGT ATT TA
HβD2 (promoter)		
−2 kb	Forward	CAT CCT TTA CTG TGA TGA TGC CC
	Reversed	AGC AGT CTA AAC CTG TGA CCA
NF-κB −221	Forward	CAC TCC ATT CAC ACA CTG GG
	Reversed	AAG AAC AAT GCA CAC TCA GGA GA

16S-based metagenomics

V5-V6	Forward	NNN NNN NAG GAT TAG ATA CCC TGG TA
	Reversed	CRR CAC GAG CTG ACG AC
Adaptor	Forward	TCGTCGGCAGCGTCAGATGTGTATAAGAGACAG
	Reversed	GTCTCGTGGGCTCGGAGATGTGTATAAGAGACAG
