# Supplementary material for: HPV infection alters vaginal microbiome through down-regulating host mucosal innate peptides used by Lactobacilli as amino acid sources
Source: Nat Commun. 2022 Feb 28;13:1076. doi: 10.1038/s41467-022-28724-8 (PMC8885657; doi:10.1038/s41467-022-28724-8)
Supplement: Supplementary file 3 — Description of the additional supplementary file [file 41467_2022_28724_MOESM3_ESM.docx]

**Description of Additional Supplementary Files**

**File name:** Supplementary Data 1

**Description:** Bacterial peptides (and related proteins) containing ^13^C_6_^15^N_2_-labeled lysines (from exogenous elafin) in their sequence. These data were obtained by mass spectrometry.
