# Supplementary material for: HPV infection alters vaginal microbiome through down-regulating host mucosal innate peptides used by Lactobacilli as amino acid sources
Source: Nat Commun. 2022 Feb 28;13:1076. doi: 10.1038/s41467-022-28724-8 (PMC8885657; doi:10.1038/s41467-022-28724-8)
Supplement: Supplementary file 5 — Reporting Summary [file 41467_2022_28724_MOESM5_ESM.pdf]

nature research

Corresponding author(s): Michael Herfs

Last updated by author(s): 2022/01/28

Reporting Summary

Nature Research wishes to improve the reproducibility of the work that we publish. This form provides structure for consistency and transparency in reporting. For further information on Nature Research policies, see Authors & Referees and the Editorial Policy Checklist .

Statistics

For all statistical analyses, confirm that the following items are present in the figure legend, table legend, main text, or Methods section.

n/a	Confirmed	
☐	☒	The exact sample size (n) for each experimental group/condition, given as a discrete number and unit of measurement
☐	☒	A statement on whether measurements were taken from distinct samples or whether the same sample was measured repeatedly
☐	☒	The statistical test(s) used AND whether they are one- or two-sided<br>*Only common tests should be described solely by name; describe more complex techniques in the Methods section.*
☐	☒	A description of all covariates tested
☐	☒	A description of any assumptions or corrections, such as tests of normality and adjustment for multiple comparisons
☐	☒	A full description of the statistical parameters including central tendency (e.g. means) or other basic estimates (e.g. regression coefficient) AND variation (e.g. standard deviation) or associated estimates of uncertainty (e.g. confidence intervals)
☐	☒	For null hypothesis testing, the test statistic (e.g. F, t, r) with confidence intervals, effect sizes, degrees of freedom and P value noted<br>*Give P values as exact values whenever suitable.*
☒	☐	For Bayesian analysis, information on the choice of priors and Markov chain Monte Carlo settings
☒	☐	For hierarchical and complex designs, identification of the appropriate level for tests and full reporting of outcomes
☒	☐	Estimates of effect sizes (e.g. Cohen's d, Pearson's r), indicating how they were calculated

Our web collection on statistics for biologists contains articles on many of the points above.

Software and code

Policy information about availability of computer code

Data collection	BD FACSDiva (version 8.0.1). No new/custom software or algorithm was used.
Data analysis	GraphPad Prism 8, FlowJo (version 10.5.3), ImageJ, MS Excel 2016, Methprimer, qPCR efficiency calculator, MOTHUR (v141.1), MassHunter Qualitative analysis (version 7.0)

For manuscripts utilizing custom algorithms or software that are central to the research but not yet described in published literature, software must be made available to editors/reviewers. We strongly encourage code deposition in a community repository (e.g. GitHub). See the Nature Research guidelines for submitting code & software for further information.

Data

Policy information about availability of data

All manuscripts must include a data availability statement. This statement should provide the following information, where applicable:

- Accession codes, unique identifiers, or web links for publicly available datasets
- A list of figures that have associated raw data
- A description of any restrictions on data availability

The source data underlying all figures are provided as a Source Data file. Any further information about resources and reagents should be directly requested to the corresponding author and will be fulfilled upon reasonable request. Raw amplicon sequencing libraries (16S-based metagenomic) that support the in vivo findings of this study have been deposited in the NCBI Sequence Read Archive (SRA) repository with the accession code: PRJNA670165. The alignment and taxonomical assignment (from phylum to species) of post-sequencing data were performed using the MOTHUR software package (v141.1), based on the SILVA database (v1.38) of full-length 16S rDNA sequences. The proteomics data have been deposited at the ProteomeXchange Consortium via the PRIDE partner repository with the following dataset identifier: PXD022113.

Field-specific reporting

Please select the one below that is the best fit for your research. If you are not sure, read the appropriate sections before making your selection.

- [x] Life sciences
- [ ] Behavioural & social sciences
- [ ] Ecological, evolutionary & environmental sciences

For a reference copy of the document with all sections, see nature.com/documents/nr-reporting-summary-flat.pdf

Life sciences study design

All studies must disclose on these points even when the disclosure is negative.

Sample size	No statistical predermination of sample size was done. All experiments were performed using sample size based on standard protocols in the field. Unless otherwise noted, n=3 biological replicates were performed for statistical significance.
Data exclusions	No data were excluded from the analysis.
Replication	The experimental findings were reliably reproduced. Data are representative of at least 3 independent experiments.
Randomization	Except for the distribution of animals (mices) in cages, no randomization was explicitly performed because the experiments were highly reproducible and independently performed by several researchers.
Blinding	No blinding was done during the experiments. The research staff was not blinded to sample allocation because the results were obtained by objective/quantitative methods of molecular and cellular biology. The experiments have been repeated by several members of the research team and reliably reproduced.

Reporting for specific materials, systems and methods

We require information from authors about some types of materials, experimental systems and methods used in many studies. Here, indicate whether each material, system or method listed is relevant to your study. If you are not sure if a list item applies to your research, read the appropriate section before selecting a response.

Materials & experimental systems

n/a	Involved in the study	
[ ]	[x]	Antibodies
[ ]	[x]	Eukaryotic cell lines
[x]	[ ]	Palaeontology
[ ]	[x]	Animals and other organisms
[ ]	[x]	Human research participants
[x]	[ ]	Clinical data

Methods

n/a	Involved in the study	
[x]	[ ]	ChIP-seq
[ ]	[x]	Flow cytometry
[x]	[ ]	MRI-based neuroimaging

Antibodies

Antibodies used

Flow cytometry (FACS): anti-CD14 PE (1/33, clone TUK4, Dako), anti-TLR4 PE (1/33, FAB6248P, R&D systems), anti-TNFR1 PE (1/33, clone W15099A, Biolegend) and anti-TNFR2 PE (1/33, clone 3G7A02, Biolegend).

Immunohistochemistry (IHC): anti-SLPI (1/400, NBP1-76803, Novus Biologicals), anti-S100A7 (1/500, HPA006997, Atlas Antibodies), anti-elafin (1/150, clone FL-117, Santa Cruz Biotechnology), anti-LL37/CAMP (1/1000, HPA029874, Atlas Antibodies), anti-HβD1 (1/50, #DEF01-A, Biologo), anti-HβD2 (1/100, ab63982, Abcam), anti-HβD3 (1/300, NB200-117, Novus Biologicals), anti-HβD4 (1/100, clone L13-10-D1, Abcam), anti-HD-5 (1/200, HPA015775, Atlas Antibodies), anti-HD-6 (1/50, NBP1-84281, Novus Biologicals), anti-p65 (1/500, clone D14E12, Cell Signaling), anti-E-cadherin (ready to use, clone 36, Ventana Medical Systems), anti-β-catenin (1/100, clone E247, Abcam), anti-c-Myc (1/100, ab32072, Abcam), anti-p16INK4a (1/300, clone JC8; Santa Cruz Biotechnology) and anti-ki67 (ready to use, clone 30-9; Ventana Medical Systems).

Western blot (WB): anti-p65 (1/1000, clone D14E12, Cell Signaling Technology), anti-IKBα (1/1000, clone L35A5, Cell Signaling Technology), anti-NEMO (1/1000, clone FL-419, Santa Cruz Biotechnology), anti-c-Myc (1/1000, ab32072, Abcam), anti-E-cadherin (1/1000, clone 36, BD Biosciences), anti-β-catenin (1/1000, clone E247, Abcam), anti-CK1 (1/1000, #2655, Cell Signaling Technology), anti-β-TrCP (1/1000, #11984, Cell Signaling Technology), anti-phospho-β-catenin (1/1000, Ser45, #9564, Cell Signaling Technology), anti-actin (1/2000, clone AC-15, Sigma Aldrich), anti-HSC-70 (1/2000, clone B-6, Santa Cruz Biotechnology), anti-NBS-1 (1/1000, BD Biosciences) and anti-MEK2 (1/1000, #9125, Cell Signaling Technology).

WB after Co-immunoprecipitation (Co-IP): anti-GLuc (1/1000, #E8023, New England Biolabs) and anti-FLAG antibody (1/2000, clone M2, Sigma Aldrich).

	Co-IP: anti-FLAG M2 magnetic beads (ready to use, Sigma Aldrich), anti-NEMO (1/50, ab178872, Abcam), anti-CK1α1 (1/30, ab206652, Abcam) and anti- β-TrCP (1/200, clone D13F10, Cell Signaling).<br><br>Chromatin immunoprecipitation (ChIP): anti-p65 antibody (1/50, clone D14E12, Cell Signaling Technology)
Validation	All antibodies are commercially available and have been validated by the manufacturers. Detailed information can be found on the website from the manufacturers. Therefore, no additional validation was performed.

Eukaryotic cell lines

Policy information about cell lines

Cell line source(s)	Lenti-X 293T cells were purchased from Clontech (Mountain View, CA, 658 USA). Immortalized keratinocytes (HaCaT cells) transduced with HPV16 E6 and/or E7 were generated in the Lab (with the help of GIGA viral vector platform). NIKS cells were generated and kindly provided by Prof Paul Lambert (University of Wisconsin). These latter cells are not commercially available.
Authentication	HPV-positive and negative keratinocytes (HaCaT and NIKS) were authenticated by cellular morphology and real-time PCR. Regarding Lenti-X-293T cells, they were not authenticated as they were purchased directly from suppliers.
Mycoplasma contamination	All cell lines used in this study have been regularly tested for potential mycoplasma contamination using the MycoAlert™ Mycoplasma Detection kit and were found to be negative.
Commonly misidentified lines (See ICLAC register)	No commonly misidentified lines were used.

Animals and other organisms

Policy information about studies involving animals; ARRIVE guidelines recommended for reporting animal research

Laboratory animals	FVB/n mice expressing HPV16 under the control of the keratin 14 promoter (K14-HPV16 transgenic mice) were obtained from the National Cancer Institute Mouse Repository. Control (FVB/n) mice were obtained from our local animal facility. For the present study, 6 to 8-week-old female mice were used. Mice were housed under standard conditions [12h light/12h dark cycle, ad libitum access to food and water, constant temperature (19-21°C) and humidity (40-50%)].
Wild animals	no wild animals were used in the study.
Field-collected samples	no field-collected samples were used in the study.
Ethics oversight	All animal experiments were performed in strict compliance with the ethical rules/recommendations established by the Federation of European Laboratory Animal Sciences Associations (FELASA) after reviewing and approval by the Institutional Ethics Committee of the University of Liege (Liege, Belgium) (#2019-60)

Note that full information on the approval of the study protocol must also be provided in the manuscript.

Human research participants

Policy information about studies involving human research participants

Population characteristics	Frozen and paraffin-embedded cervical/vulvar specimens were retrieved from the Tissue Biobank of the University Hospital Center of Liege (Belgium). All patients underwent a surgical procedure between 2011 and 2015.
Recruitment	The tissue specimens were selected based on their histopathological diagnoses (normal, dysplasia, invasive carcinoma). Before being used in this study, the original diagnoses were re-examined by senior histopathologists and, to avoid misclassification (potential bias), both the proliferative index (nuclear Ki67 staining) and p16INK4a expression of each specimen were determined by immunohistochemistry.
Ethics oversight	A signed informed consent was directly obtained by the local Biobank. Before being used for research purposes, all tissue specimens were anonymized by the Biobank. The use of both frozen and paraffin-embedded tissues was approved by the Ethics Committee of the University Hospital of Liege (Belgium).

Note that full information on the approval of the study protocol must also be provided in the manuscript.

Flow Cytometry

Plots

Confirm that:

- [x] The axis labels state the marker and fluorochrome used (e.g. CD4-FITC).
- [x] The axis scales are clearly visible. Include numbers along axes only for bottom left plot of group (a 'group' is an analysis of identical markers).
- [ ] All plots are contour plots with outliers or pseudocolor plots.
- [ ] A numerical value for number of cells or percentage (with statistics) is provided.

Methodology

Sample preparation	Cells were scraped and washed twice with PBS 1X before adding the primary antibody for 30 minutes at 4°C. After washing/centrifugation, cells were fixed with 1% paraformaldehyde before flow cytometry analysis.
Instrument	FACS Canto II
Software	FlowJo (version 10), BD FACSDiva (version 6.1.2) softwares.
Cell population abundance	A total of 10,000 live, single cells were recorded for each sample.
Gating strategy	For all FACS analyses, the gating strategy used was forward and side scatter gating — to remove debris and other events of noninterest while preserving cells based on size and/or complexity.

- [x] Tick this box to confirm that a figure exemplifying the gating strategy is provided in the Supplementary Information.
